# Ketogenic diet therapies for the treatment of drug-resistant epilepsy in children and adults: A systematic review

**DOI:** 10.1371/journal.pone.0333334

**Published:** 2026-07-02

**Authors:** María Magdalena Vaccarezza, Verónica Laura Sanguine, Giselle Balaciano

**Affiliations:** 1 Italian Hospital of Buenos Aires, Ciudad Autónoma de Buenos Aires, Argentina; 2 National Ministry of Health of Argentina, Ciudad Autónoma de Buenos Aires, Argentina; Aga Khan University Hospital, PAKISTAN

## Abstract

Epilepsy is a common treatable neurological condition characterized by recurrent involuntary brain activity manifested in seizures. It is estimated that around 30% of patients with this disease do not respond to initial pharmacological treatments, developing drug-resistant epilepsy. Among the non-pharmacological treatment options are ketogenic diet therapies (KDT) in its various forms. The objective of this study is to systematically review the randomized controlled trials investigating the use of KDTs in pediatric and adult drug-resistant epilepsy, according to Preferred Reporting Items for Systematic Review and Meta-analysis (PRISMA) guidelines. The following databases: Embase, PubMed/Medline, LILACS, and the Cochrane Library, were searched and studies fitting the inclusion and exclusion criteria were included for analysis. Randomized controlled trials (RCT) with a minimum follow-up of 28 days were included. There were 1193 articles retrieved after duplicates were removed and 17 met the inclusion criteria. Eleven studies included children (up to 12 yrs) and six included adolescents from 13 years old and adults. Follow-up ranged from 6 to 24 months. In children, 37% may achieve a reduction in seizure frequency of 50% or more with in any form of KDT (moderate-certainty evidence). In addition, about 6 more children per 100 may achieve a ≥ 90% reduction, although this is supported by low-certainty evidence. In adolescents and adults, KDT may lead to a ≥ 50% reduction in seizure frequency in 16 more individuals per 100 compared with usual care (moderate-certainty evidence), but its impact on a 90% or greater reduction is uncertain due to the limited number of reported events and imprecision in available studies. Side effects in children showed no significant differences compared to usual care (low certainty), while in adults, the impact remains uncertain (very low certainty). Adherence to treatment may be slightly lower with KDT in both children and adults/adolescents compared to usual treatment, though results are inconsistent. Regarding quality of life and cognitive and behavioral outcomes, studies are scarce, heterogeneous, and of very low certainty, limiting the ability to draw strong conclusions.

## 1. Introduction

Epilepsy is a common and treatable neurological disorder characterized by recurrent episodes of involuntary brain activity, clinically manifested as seizures [1,2]. It affects approximately 50 million people worldwide, with an estimated 30% of patients developing drug-resistant epilepsy, defined as the failure to respond to 2 or 3 appropriately chosen antiseizure medication. This form of epilepsy is associated with a poor prognosis and significantly impaired quality of life [3,4].

Among the non-pharmacological treatment options available, are ketogenic diet therapies (KDTs), in their different forms [5]. These dietary therapies typically consist of high fat, low carbohydrate, and adequate protein content, with the specific macronutrient ratios determining the type of KDT employed [6,7]. The selection of an appropriate KDT depends on several factors, including the etiology and type of epilepsy, patient age, and individual and family characteristics [8].

There are different types of KDTs, the most commonly used in children are the classical ketogenic diet (CKD) and the medium-chain triglyceride (MCT) ketogenic diet, which provides 30–60% of daily energy requirements through MCTs(6, 7). The modified Atkins diet [9] is characterized by a significant reduction in carbohydrate intake and the fat/protein and carbohydrate ratio is estimated at an average of 1:1, with protein intake generally unrestricted [10,11]. The low glycemic index (LGI) ketogenic diet is yet another approach designed to be more practical and sustainable, allowing a more liberal intake of carbohydrates with a focus on those with a low glycemic index [12].

The biochemical mechanisms underlying the anticonvulsant effects of KDT are not fully understood. Several theories have been proposed, including the direct anticonvulsant action of ketone bodies and/or fatty acids, as well as a reduction in circulating glucose levels [13]. Ketone bodies may upregulate the expression of genes involved in mitochondrial biogenesis in neurons and glial cells, potentially enhancing cellular energy metabolism and reducing seizure susceptibility [14–16].

A systematic review published by the Cochrane Collaboration in 2020 [17] concluded that KDT could be an effective treatment for children with refractory epilepsy, with low to very low certainty in the evidence available to date. In adult patients, the evidence was very limited and of very low certainty, making it impossible to draw conclusions. In recent years, new studies on this intervention have been published, creating the need for this review to improve the certainty of the previously described results.

## 2. Methods

A systematic review was performed according to PRISMA statement [18] (Preferred Reporting Items for Systematic Reviews and Meta-analysis) guidelines and an a priori protocol was registered in PROSPERO under the number CRD42024516319.

### 2.1. Eligibility criteria

The Population, Intervention, Comparator and Outcome (PICO) framework was used to define the inclusion criteria and to formulate the research question.

Studies were eligible for inclusion if they were randomized controlled trials (RCTs) that investigated any form of ketogenic diet therapy (KDT) as an intervention, had a minimum duration of 28 days, and included pediatric or adult patients diagnosed with drug-resistant epilepsy, irrespective of epilepsy type or syndrome.

Studies were excluded if the intervention period was shorter than 28 days or if both the intervention and control groups received different forms of ketogenic therapy.

### 2.2. Search strategy

Electronic searches were conducted in PubMed, Embase, the Cochrane Library and LILACS in February 2024 and updated in June 2025. The search strategy combined controlled vocabulary and free-text synonyms related to drug-resistant epilepsy and ketogenic diets. Studies published in English, Spanish, French, and Portuguese were considered, with no restrictions on publication date. We also sought to identify additional evidence using a range of methods: expert consultation, citation tracking, and screening reference lists of studies identified through the database searches. The full search strategy is provided in the Supplementary Material (S1 Appendix).

### 2.3. Article selection

Electronic database search results were downloaded into a reference management system (EndNote™) and subsequently imported into the Rayyan tool [9] for screening. Duplicates were removed before screening the title and abstract of all results for eligibility against the inclusion and exclusion criteria. Screening was undertaken by two independent reviewers (GB and VS) in an independent and blinded fashion. In cases of disagreement or uncertainty regarding study eligibility, a consensus was reached through consultation with a third reviewer (MV).

### 2.4. Data extraction, analysis and assessment of bias

Two reviewers independently applied predefined criteria to extract data and assess the methodological quality of the included studies. The following outcomes were evaluated: overall survival, seizure freedom or a reduction in seizure frequency of 90% or greater, seizure reduction (defined as a 50% or greater reduction in seizure frequency), side effects, cognition and behavior, quality of life, dropout rate, hospitalizations, status epilepticus, and survival.

An intention-to-treat (ITT) approach was used for all analyses. Results are presented as relative risks (RR) with corresponding 95% confidence intervals (CI). Where appropriate, a meta-analysis was conducted using Review Manager (RevMan) 5.4.1 software. Due to substantial heterogeneity, DerSimonian and Laird random-effects models were used. Outcomes related to cognition, behavior, and quality of life were analyzed narratively due to their qualitative nature.

Statistical heterogeneity was evaluated using the I² statistic and the chi-square (Q) test.

The risk of bias in individual studies was assessed using the Cochrane Risk of Bias 2 tool [19] (RoB 2). The certainty of the evidence for each outcome was evaluated using the GRADE (Grading of Recommendations Assessment, Development and Evaluation) approach [20].

## 3. Results

### 3.1. Search results and included studies

**Fig 1:**
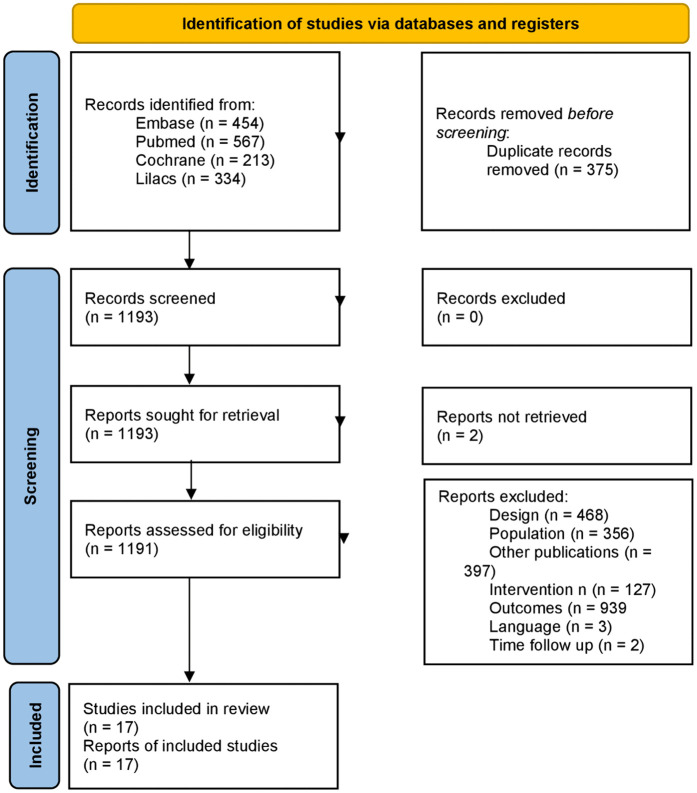
PRISMA Flowchart of included studies.

The search was conducted in February 2024 and updated in June 2025, where 1568 studies were retrieved from electronic databases. After eliminating duplicates, 1193 studies were screened by title and abstract. Finally, 17 studies were analyzed by full text and included in this review. (Fig 1)

### 3.2. Included studies

Of the 17 included studies, 6 were in adults and adolescents [21–26], and 11 in children [27–37]. The duration ranged from six to 24 months. All of them compared some form of KDT with usual care for the underlying condition, for example, an additional line of medication. A summary of the characteristics of the included studies can be seen in Tables 1 and 2.

**Table 1:** Characteristics of included studies for children.

Author & Year	Title	Design	Population	Intervention & Comparator	Main Outcomes	Follow-up
Archna et al., 2022	Efficacy of Modified Atkins Diet [9] vs. Levetiracetam in Refractory Epilepsy	RCT	101 children (mean age 60.1 and 62.5 months) with drug-resistant	MAD vs. Levetiracetam	≥50% reduction in seizure frequency ≥ 90% reduction in seizure frequency Side effects Dropout rate	12 weeks
Dressler et al., 2019	Comparison of Ketogenic Diet (KD) vs. ACTH in West Syndrome	RCT	101 children (mean age 4.9 and 5.0 months) with West syndrome	KD vs. ACTH	≥90% reduction in seizure frequency Side effects Dropout rate Cognition and behaviour	24 months
Lambrechts et al., 2016	A randomized controlled trial of the ketogenic diet in refractory childhood epilepsy	RCT	57 children and adolescents (mean age 2.4 and 1.9 years) with drug-resistant epilepsy	KD or MCT vs. SC	≥50% reduction in seizure frequency ≥ 90% reduction in seizure frequency Dropout rate	4 months
El-Rashidy et al., 2013	Modified Atkins Diet vs. Classic Ketogenic Diet in Refractory Childhood Epilepsy	RCT	40 children (inclusion criteria 12–36 months) with drug-resistant epilepsy	KD vs. MAD vs. SC	Seizure frequency	6 months
Neal et al., 2008	The Ketogenic Diet for the Treatment of Childhood Epilepsy: A Randomized Controlled Trial	RCT	145 children (inclusion criteria 2–16 years) with drug-resistant epilepsy	KD or MCT vs. SC	≥50% reduction in seizure frequency ≥ 90% reduction in seizure frequency Dropout rate	3 months
Sharma et al., 2021	Evaluation of Modified Atkins Diet in Hormone Therapy-Resistant Epileptic Spasms	RCT	91 children (mean age 21.9 and 18.6 months) with hormone therapy-resistant epileptic spasms	MAD vs. SC	≥50% reduction in seizure frequency ≥ 90% reduction in seizure frequency Dropout rate	4 weeks
Sharma et al., 2013	Use of Modified Atkins Diet for Refractory Childhood Epilepsy	RCT	102 children (mean age 4.7 to 5.2 years) with drug-resistant epilepsy	MAD vs. SC	≥50% reduction in seizure frequency ≥ 90% reduction in seizure frequency Dropout rate	3 months
Sharma et al., 2016	Evaluation of a Simplified Modified Atkins Diet for Refractory Epilepsy	RCT	81 children (mean age 5.6 and 4.8 years) with drug-resistant epilepsy	MAD vs. SC	≥50% reduction in seizure frequency ≥ 90% reduction in seizure frequency Dropout rate	3 months
Lakshminarayanan et al., 2021	Efficacy of Low Glycemic Index Therapy in Children Aged 2–8 Years with Drug-Resistant Epilepsy: A Randomized Controlled Trial	RCT	40 children (mean age 3.85 and 3.95 years) with drug-resistant epilepsy	LGIT vs. SC	≥50% reduction in seizure frequency ≥ 90% reduction in seizure frequency Side effects	3 months
Huang et al., 2022	Efficacy and Safety of the Ketogenic Diet in Mitochondrial Diseases with Epilepsy: A Prospective Controlled Study	RCT	33 children (mean age 79 and 76 months) with mitochondrial disease-associated epilepsy	KD vs. SC	≥50% reduction in seizure frequency ≥ 90% reduction in seizure frequency Side effects Dropout rate	3 months
Schoeler et al., 2023	Classic Ketogenic Diet vs. Additional Antiseizure Medication in Infants with Drug-Resistant Epilepsy (KIWE)	RCT	136 children (inclusion criteria 1–24 months)_ with drug-resistant epilepsy	KD vs. additional antiseizure medication	≥50% reduction in seizure frequency ≥ 90% reduction in seizure frequency Side effects Dropout rate Cognition and behaviour Quality of life	11.3 months

RCT: Randomized Controlled Trial.

KD: Ketogenic Diet.

LGIT: Low Glycemic Index Treatment.

SC: Standard Care.

MAD: Modified Atkins Diet.

MCT: Medium-Chain Triglyceride.

ACTH: Adrenocorticotropic Hormone.

**Table 2:** Characteristics of included studies for adults and adolescents.

Author & Year	Title	Design	Population	Intervention & Comparator	Main Outcomes	Follow-up
Kishk et al., 2021	Effect of the Ketogenic Diet in Adolescents and Adults with Drug-Resistant Epilepsy	RCT	80 adolescents and adults with drug-resistant epilepsy	KD vs. SC	≥50% reduction in seizure frequency	3 months
Borges et al., 2019	Randomized Trial of Triheptanoin vs. Medium-Chain Triglycerides as Adjunctive Therapy in Refractory Epilepsy	RCT	34 adults with drug-resistant epilepsy	Triheptanoin vs. MCT	≥50% reduction in seizure frequency Side effects Dropout rate	18 weeks
Kverneland et al., 2023	Health-Related Quality of Life in Adults with Drug-Resistant Focal Epilepsy Treated with the Modified Atkins Diet	RCT	39 adults with drug-resistant focal epilepsy	MAD vs. SC	Quality of life	12 weeks
Kverneland et al., 2018	Effect of the Modified Atkins Diet in Adults with Drug-Resistant Focal Epilepsy	RCT	75 adults with drug-resistant epilepsy	MAD vs. SC	≥50% reduction in seizure frequency ≥ 90% reduction in seizure frequency Side effects Dropout rate	12 weeks
Manral et al., 2023	Safety, Efficacy, and Tolerability of the Modified Atkins Diet in People with Drug-Resistant Epilepsy	RCT	160 adults with drug-resistant epilepsy	MAD vs. SC	≥50% reduction in seizure frequency ≥ 90% reduction in seizure frequency Side effects Dropout rate Quality of life	6 months
Zare et al., 2017	Efficacy of the Modified Atkins Diet in Adults with Refractory Epilepsy	RCT	66 adults with drug-resistant epilepsy	MAD vs. SC	≥50% reduction in seizure frequency ≥ 90% reduction in seizure frequency Dropout rate	2 months

RCT: Randomized Controlled Trial.

KD: Ketogenic Diet.

SC: Standard Care.

MAD: Modified Atkins Diet.

MCT: Medium-Chain Triglyceride.

### 3.3. Risk of bias in included studies

**Fig 2:**
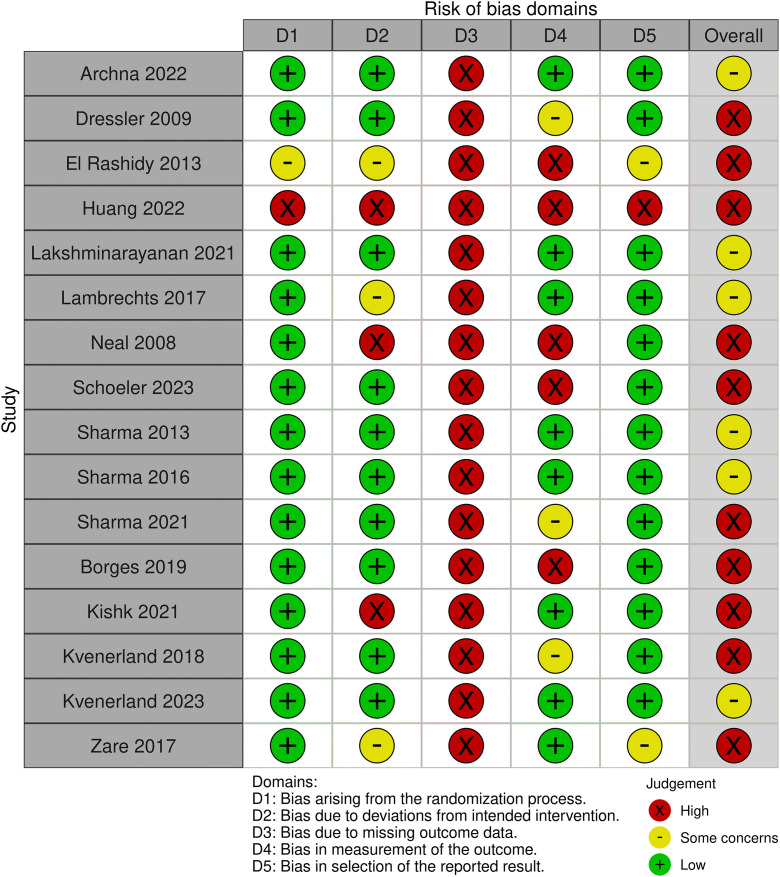
Summary of risk of bias.

Overall, sequence generation and allocation concealment were uniformly robust. However, blinding—particularly of outcome assessment—was a frequent weakness, as it was not possible due to the nature of the intervention (diet versus medication). Incomplete data and other biases were generally well handled, and no evidence of selective reporting was detected. (Fig 2)

Results for adults and children are reported separately.

### 3.4. Heterogeneity

The heterogeneity was considerable for some outcomes.

It was suggested that this could be due to the different age groups included in the studies on children. These variables were explored but no single determinant was found.

We used the ICEMAN tool [38] to assess the possible modifying effect of age (> or <than 1 year). The result showed that the credibility of considering age greater or less than one year as an effect modifier is low. Therefore, it was decided to present the data by including all studies in a single meta-analysis.

### 3.5. Effect of the interventions

Results are reported separately for children (Table 3), and adults and adolescents (Table 4)

**Table 3:** Summary of findings table of ketogenic diet compared to usual care for drug-resistant epilepsy in children.

Outcome	No. of participants (studies)	Relative effect (95% CI)	Anticipated absolute effects (95% CI)			Certainty	What happens
**Without ketogenic diet**	**With ketogenic diet**	**Difference**
≥50% seizure reduction	786 (9 RCTs)	RR 4.06 (1.97 to 8.37)	12.2%	49.7% (24.1 to 100)	37.4% more (11.9 more to 90.2 more)	⨁⨁⨁◯ Moderate a,b	37 more children out of 100 likely reduce seizures by ≥50% (95% CI 12–90 more)
≥90% seizure reduction	759 (10 RCTs)	RR 1.74 (1.00 to 3.03)	8.3%	14.4% (8.3 to 25)	6.1% more (0 fewer to 16.8 more)	⨁⨁◯◯ Low a,c	6 more children out of 100 may reduce seizures by ≥90% (95% CI 0–17 more)
Adverse events	342 (5 RCTs)	RR 1.03 (0.77 to 1.39)	39.4%	40.5% (30.3 to 54.7)	1.2% more (9.1 fewer to 15.3 more)	⨁⨁◯◯ Low a,d	Ketogenic diet may make little or no difference in adverse events
Dropout rate	818 (10 RCTs)	Not pooled		11.4%	Not pooled	⨁⨁◯◯ Low a,c	Ketogenic diet may slightly increase dropout rate
Quality of life	217 (2 RCTs)	--		--		⨁◯◯◯ Very low a,d	Effect is uncertain
Psychomotor development – cognitive aspects	146 (3 RCTs)	--		--		⨁◯◯◯ Very low a,d	Effect is uncertain

Explanations:

a. Unblinded studies, risk of co-interventions.

b. Although I² > 65%, most studies are consistent, so no rating down for heterogeneity.

c. 95% CI crosses/reaches the line of no effect.

d. 95% CI crosses the line of no effect; optimal information size not reached.

**Table 4:** Summary of findings table of ketogenic diet compared to usual care for drug-resistant epilepsy in adults and adolescents.

Outcome	No. of participants (studies)	Relative effect (95% CI)	Anticipated absolute effects (95% CI)			Certainty	What happens
			Without ketogenic diet	With ketogenic diet	Difference		
≥50% seizure reduction	414 (5 RCTs)	RR 7.73 (2.32 to 25.70)	2.4%	18.8% (5.6 to 62.4)	16.3% more (3.2 more to 60 more)	⨁⨁⨁◯ Moderate a,b	16 more adults/adolescents out of 100 likely reduce seizures by ≥50% (95% CI 3.2 to 60 more)
≥90% seizure reduction	301 (3 RCTs)	RR 13.00 (0.74 to 226.98)	0.0%	0.0% (0–0)	0.0% less (0 less to 0 less)	⨁◯◯◯ Very low a,c,d	Effect is uncertain
Adverse events	194 (2 RCTs)	RR 1.65 (0.15 to 18.70)	14.4%	23.8% (2.2 to 100)	9.4% more (12.3 fewer to 255.5 more)	⨁◯◯◯ Very low a,d,e	Effect is uncertain
Dropout rate	374 (5 RCTs)	RR 1.78 (0.83 to 3.83)	17.1%	30.5% (14.2 to 65.5)	13.3% more (2.9 fewer to 48.4 more)	⨁◯◯◯ Very low a,e,f	Effect is uncertain
Quality of life	(2 RCTs)	--		--		⨁◯◯◯ Very low a,g,h	Effect is uncertain

Explanations:

a. Unblinded studies with high risk of co-interventions.

b. Although wide CI, it does not cross statistical significance.

c. Not assessable due to low number of events.

d. Few events, optimal information size not reached.

e. I^2^ 66%.

f. Crosses the line of no statistical effect.

g. One study shows significant difference, the other does not.

h. Although mean difference is clinically relevant, reported Standard Deviations are imprecise.

#### 3.5.1. 50% or greater reduction in seizure frequency.

##### 3.5.1.1. Children.

In children, 9 studies reported this outcome [27,30–33,35–37], with a total of 786 participants. Results showed a reduction in seizure frequency by 50% or more: RR 4.06 (95% CI 1.97 to 8.37), with moderate certainty in the evidence due to a serious risk of bias in the included studies. Although the I^2^ value reflecting heterogeneity was high, it was considered that the vast majority of included studies were consistent. Therefore, although heterogeneity is noted, the certainty was not downgraded for this reason. In absolute terms, this means that with KDT, an additional 37 children per 100 on average are likely to reduce seizure frequency by 50% or more compared to usual care (95% CI 12–90 more).

##### 3.5.1.2. Adults and Adolescents.

In adults and adolescents, 5 studies reported this outcome [21–23,25,26], including a total of 494 patients. The meta-analysis result showed a RR of 7.73 (95% CI 2.32 to 25.70), with moderate certainty in the evidence given the risk of bias in the included studies. In absolute terms, with KDT, an additional 16 adults or adolescents per 100 on average are likely to reduce seizures by 50% or more compared usual care (95% CI 3.2 to 60).

#### 3.5.2. 90% or more reduction in seizures frequency (including seizure freedom).

##### 3.5.2.1. Children.

In children, 10 studies reported this outcome [27,28,30–37], including data from 759 participants. The meta-analysis result showed a RR of 1.74 (95% CI 1.00 to 3.03), with low certainty in the evidence due to a serious risk of bias in the included studies as well as imprecision as the result crosses the line of no statistical difference. In absolute terms, with the KDT, an additional 6 children per 100 could reduce seizure frequency by 90% or more on average (95% CI 0–17 more).

##### 3.5.2.2. Adults and Adolescents.

In adults and adolescents, 3 studies reported this outcome [23,25,26], including a total of 301 patients. The meta-analysis result showed an RR of 13.00 (95% CI 0.74 to 226.98). Given the low number of studies reporting on this outcome (only the intervention arm of Manral’s [25] study reported events, while the other studies did not report events in any of their arms) and the assessed risk of bias, it is concluded that there is uncertainty about the impact of KDT on this outcome in adults and adolescents.

#### 3.5.3. Side Effects.

##### 3.5.3.1. Children.

In children, 5 clinical trials [27,28,30,31,34] with a total of 342 participants reported the outcome of side effects as the number of individuals experiencing at least one adverse event. The RR was 1.03 (95% CI 0.77 to 1.39) with low certainty in the evidence due to a risk of bias in the included studies and imprecision (the 95% CI includes the measure of no effect). In absolute terms, the use of KDT in children may not have significant differences compared to usual care regarding side effects.

##### 3.5.3.2. Adults and Adolescents.

In adults and adolescents, 3 studies reported this outcome [21,23,25], including a total of 194 patients. The meta-analysis result showed an RR of 1.65 (95% CI 0.15 to 18.70), which represents 94 more side effects per 1000 patients (95% CI 123 fewer to 1000 more) in the intervention group. Due to the risk of bias, inconsistency in the results (I2 = 66%), and imprecision (the confidence interval includes both benefits and harms), the certainty in the evidence for this outcome was rated as very low. The main side events reported by the studies were gastrointestinal (vomiting, diarrhea, nausea, reflux, constipation, abdominal pain, and weight loss), which resolved upon discontinuation of the diet. Thus, it is concluded that the impact of KDT on side effects in adults and adolescents is uncertain.

#### 3.5.4. Dropout Rate.

##### 3.5.4.1. Children.

In children, 10 studies [27,28,30,32–37] including 818 participants reported this outcome, with follow-up ranging from 4 weeks to 24 months. The conducted meta-analysis estimated an RR of 1.64 (95% CI 0.88 to 3.07). This can be interpreted in absolute terms as indicating that KDT might slightly increase the dropout rate compared to standard treatment. The certainty in the evidence was rated as low due to the risk of bias in the included studies and serious imprecision.

##### 3.5.4.2. Adults and Adolescents.

In adults and adolescents, 5 studies reported this outcome [21,23–26], including a total of 374 patients. The meta-analysis result showed an RR of 1.78 (95% CI 0.83 to 3.83), which represents, on average, 133 more patients per 1000 dropping out (95% CI 29 fewer to 484 more) in the intervention group. The certainty in the evidence for this outcome was rated as very low due to risk of bias, inconsistency in the results (I2 = 66%), and imprecision (the confidence interval includes both benefits and harms). Therefore, the impact of KDT on dropout rates in adults and adolescents is uncertain.

#### 3.5.5. Quality of Life.

##### 5.5.5.1. Children.

Two studies [34,35] reported this outcome in children in a structured manner. No meta-analysis was conducted due to differences in scales, measurement, and reporting. Results are described narratively:

In Schoeler´s study [34], no differences were observed between groups for any ITQOL-97 domain at 12 months, except for the child’s temperament and mood (β coefficient –6.09, 95% CI –11.63 to –0.54) and peer relationships (β coefficient –6.79, –12.97 to –0.60), which favored the anticonvulsant medication group.In Sharma’s study [35], parents and caregivers in the intervention group reported improvements in alertness (66.6%), activity level (58.3%), sleep (72.2%), social interaction (52.7%), and behavior (52.7%) with KDT compared to usual care.

## 4. Discussion

### 4.1. Summary of main results

This systematic review evaluated the effectiveness and safety of ketogenic diet therapies (KDs) in children, adolescents, and adults with drug-resistant epilepsy. The evidence suggests that KDs may provide a clinically meaningful benefit in reducing seizure frequency, particularly for moderate reductions. This benefit appears more consistent in children, with a similar trend observed in adults and adolescents. The evidence for substantial seizure reduction, such as near-complete control, is still emerging, especially in adult populations. Side effects may not differ substantially from usual care in children, but the impact in adults and adolescents is highly uncertain. The most common adverse events were gastrointestinal and generally resolved after discontinuing the diet. Adherence is a particularly relevant issue in dietary interventions. Although the certainty of evidence was low in children and very low in adults, our findings suggest that dropout rates from KD may not differ significantly from those of standard treatments.

The evidence on quality of life, cognitive, and behavioral outcomes, hospitalization rates and overall survival was sparse and inconclusive. Future locally generated evidence could provide high certainty evidence on adverse events, quality of life, and dropout rates.

### 4.2. Overall completeness and applicability of the evidence

The included studies provided data across a range of ketogenic diet types and populations. However, most studies were conducted in high-income countries, which may limit generalizability to low- and middle-income settings where dietary patterns, resources, and healthcare infrastructures differ. The applicability of results to subgroups, such as infants or individuals with specific epilepsy syndromes, remains uncertain due to limited subgroup data and inconsistent reporting.

### 4.3. Quality/certainty of the evidence

The overall certainty of evidence ranged from moderate for seizure reduction to low or very low for other outcomes, including seizure freedom, side effects, adherence, and quality of life. The primary factors leading to downgrading were risk of bias (mainly due to lack of blinding and potential for co-interventions), imprecision (due to small numbers of events and wide confidence intervals), and, in some cases, inconsistency between studies.

### 4.4. Potential biases in the review process

We aimed to minimize bias through a comprehensive search strategy, inclusion of studies in multiple languages, and independent screening and data extraction by multiple reviewers. Nonetheless, the possibility of publication bias cannot be ruled out, as unpublished data were difficult to identify. Additionally, the absence of individual patient data limited our ability to perform detailed subgroup analyses.

### 4.5. Agreements and disagreements with other studies or reviews

Our findings are consistent with those of a prior Cochrane review published in 2020 [17], which concluded that KDs may be effective in reducing seizures in children with refractory epilepsy but noted low to very low certainty in the evidence.

That review highlighted the lack of robust data for adults, which remains a limitation despite the inclusion of newer studies in our review. Our synthesis supports and updates those conclusions, emphasizing the needs for more high level evidence related to safety, adherence, and patient-centered outcomes.

## 5. Conclusion

This systematic review evaluated the effectiveness and safety of ketogenic diet therapies (KDTs) in adults, adolescents, and children with drug-resistant epilepsy.

In children, KDTs may achieve a reduction in seizure frequency of 50% or more and may also reduce seizure frequency by 90% or more including seizure freedom, without increasing the frequency of adverse events or dropout rates compared to usual care. However, there is uncertainty regarding their impact on quality of life, psychomotor development, and cognitive outcomes.

In adults and adolescents, KDTs may reduce seizure frequency by 50% or more; however, their effects on achieving a 90% or greater seizure reduction, as well as on adverse events, dropout rates, and quality of life, remain uncertain.

No evidence was identified regarding hospitalization rates, status epilepticus, or overall survival.

## Supporting information

S1 Appendix Supporting information. (DOCX)
