# Supplementary material for: Ketogenic diet therapies for the treatment of drug-resistant epilepsy in children and adults: A systematic review
Source: PLoS One. 2026 Jul 2;21(7):e0333334. doi: 10.1371/journal.pone.0333334 (PMC13327284; doi:10.1371/journal.pone.0333334)
Supplement: S1 Appendix — (DOCX) [file pone.0333334.s001.docx]

# Supplemental material: search strategy

| Database: Embase | | | |
| --- | --- | --- | --- |
| URL: www.embase.com | | | |
| Date Searched: 19.02.2024 | | | |
| **#** | **Searches** | **Results** | **Column1** |
| 1 | refractory epilepsy'/exp OR 'epilepsy'/exp OR ‘Drug-Resistant Epileps*’:ti,ab OR ‘Medication-Resistant Epileps*’:ti,ab OR ‘Intractable Epileps*’:ti,ab OR ‘Refractory Epileps*’:ti,ab OR ‘Epileptic Syndrom*’:ti,ab OR ‘Epilepsy Syndrom*’:ti,ab OR ‘Syndromic Epilep*’:ti,ab OR ‘Familial Epileps*’:ti,ab OR 'lennox gastaut syndrome'/exp OR ‘Lennox-Gastaut Syndrome*’:ti,ab OR 'landau kleffner syndrome'/exp OR ‘Landau-Kleffner’:ti,ab OR ‘Acquired Aphasia’:ti,ab OR 'infantile spasm'/exp OR ‘Spasm’:ti,ab OR ‘Seizure’:ti,ab OR 'myoclonus epilepsy'/exp OR ‘Myoclonic Epileps*’:ti,ab OR 'tuberous sclerosis'/exp OR ‘Bourneville’:ti,ab OR ‘Cerebral Scleros*’:ti,ab OR ‘Epiloia’:ti,ab OR ‘Tuberous Sclerosis’:ti,ab | 369.988 |  |
| 2 | ketogenic diet'/exp AND 'ketogen*':ti,ab OR 'medium-chain triglyceride':ti,ab OR 'mct diet*':ti,ab OR 'modified atkins':ti,ab OR 'low glycemic':ti,ab | 9665 |  |
| 3 | #2 AND #3 | 3263 |  |
| 4 | Filter: Trials | 454 |  |

| Database: Pubmed | | |
| --- | --- | --- |
| URL: www.pubmed.gov | | |
| Date Searched: 19.02.2024 | | |
| **#** | **Searches** | **Results** |
| 1 | "Drug Resistant Epilepsy"[Mesh] OR "Epileptic Syndromes"[Mesh] OR "Drug-Resistant Epileps*"[tiab] OR "Medication-Resistant Epileps*"[tiab] OR "Intractable Epileps*"[tiab] OR "Refractory Epileps*"[tiab] OR "Epileptic Syndrom*"[tiab] OR "Epilepsy Syndrom*"[tiab] OR "Syndromic Epilep*"[tiab] OR "Familial Epileps*"[tiab] OR "Lennox Gastaut Syndrome"[Mesh] OR "Lennox-Gastaut Syndrome*"[tiab] OR "Landau-Kleffner Syndrome"[Mesh] OR "Landau-Kleffner"[tiab] OR "Acquired Aphasia"[tiab] OR "Spasms, Infantile"[Mesh] OR "Spasm"[tiab] OR "Seizure"[tiab] OR "Epilepsies, Myoclonic"[Mesh] OR "Myoclonic Epileps*"[tiab] OR "Tuberous Sclerosis"[Mesh] OR "Bourneville"[tiab] OR "Cerebral Scleros*"[tiab] OR "Epiloia"[tiab] OR "Tuberous Sclerosis"[tiab] | 136.626 |
| 2 | "Diet, Ketogenic"[Mesh] OR "ketogen*"[tiab] OR "Medium-Chain Triglyceride"[tiab] OR "MCT diet*"[tiab] OR "modified atkins"[tiab] OR "low glycemic"[tiab] | 10.253 |
| 3 | #1 AND #2 | 1.853 |
| 4 | (randomized controlled trial[pt] OR controlled clinical trial[pt] OR randomized[tiab] OR placebo[tiab] OR drug therapy[sh] OR randomly[tiab] OR trial[tiab] OR groups[tiab]) NOT (animals [mh] NOT humans [mh]) | 5.218.815 |
| 5 | #3 AND #4 | 567 |

| Database: Cochrane | | |
| --- | --- | --- |
|  | | |
| Date Searched: 19.02.2024 | | |
| **#** | **Searches** | **Results** |
| 1 | MeSH descriptor: [Drug Resistant Epilepsy] explode all trees | 239 |
| 2 | MeSH descriptor: [Epileptic Syndromes] explode all trees | 588 |
| 3 | MeSH descriptor: [Lennox Gastaut Syndrome] explode all trees | 72 |
| 4 | MeSH descriptor: [Landau-Kleffner Syndrome] explode all trees | 3 |
| 5 | MeSH descriptor: [Spasms, Infantile] explode all trees | 116 |
| 6 | MeSH descriptor: [Epilepsies, Myoclonic] explode all trees | 116 |
| 7 | Drug-Resistant Epileps* OR Medication-Resistant Epileps* OR Intractable Epileps* OR Refractory Epileps* OR Epileptic Syndrom* OR Epilepsy Syndrom* OR Syndromic Epilep* OR Familial Epileps* OR Lennox-Gastaut Syndrome* OR Landau-Kleffner OR Acquired Aphasia OR Spasm OR Seizure OR Myoclonic Epileps* OR Bourneville OR Cerebral Scleros* OR Epiloia OR Tuberous Sclerosis | 14419 |
| 8 | #1 OR #2 OR #3 OR #4 OR #5 OR #6 OR #7 | 14542 |
| 9 | MeSH descriptor: [Diet, Ketogenic] explode all trees | 200 |
| 10 | ketogen* OR Medium-Chain Triglyceride OR MCT diet* OR modified atkins OR low glycemic | 8180 |
| 11 | #9 OR #10 | 8180 |
| 12 | #8 AND #11 | 304 |
| 13 | Filter: Trials | 213 |

| Database: Lilacs | | |
| --- | --- | --- |
| URL: | | |
| Date Searched: 19.02.2024 | | |
| **#** | **Searches** | **Results** |
| 1 | mh: "Epilepsies, Myoclonic" OR (Dravet Syndrom$ OR Epileps$ Infantile Myoclonic OR Early Childhood Epilepsy, Myoclonic OR Early Childhood, Myoclonic Epilepsy OR Doose Syndrome OR Infantile Myoclonic Epilep$ OR Myoclonic Seizure Disorder$ OR Myoclonus Epileps$) | 3664 |
| 2 | mh: "Drug Resistant Epilepsy" OR (Drug Refractory Epileps$ OR Drug Resistant Epilepsies OR Epileps$, Intractable OR Epilepsy, Medication Resistant) | 6166 |
| 3 | mh: "Tuberous Sclerosis" OR (Bourneville Disease$ OR Bourneville Phacomatosis OR Bourneville Phakomatosis OR Cerebral Sclerosis OR Epiloia OR Sclerosis Tubers$ OR Tuberous Sclerosis Complex) | 6819 |
| 4 | mh:"Lennox Gastaut Syndrome" OR Lennox-Gastaut Syndrome |  |
| 5 | mh:"Epileptic Syndromes" OR (Epilepsy Syndrom$ OR Familial Epileps$ OR Syndromic Epileps$) |  |
| 6 | mh:"Landau-Kleffner Syndrome" OR (Acquired Epileptic Aphasia OR Acquired Childhoood Aphasia with Convulsive Disorder OR Landau Kleffner Acquired Epileptiform Aphasia) |  |
| 7 | mh:"Diet, Ketogenic" OR (High Protein Low Carbohydrate OR Carbohydrate Restricted High Protein OR High-Protein Carbohydrate-Restricted OR High-Protein Low-Carbohydrate OR Atkins) | **11,266** |
| 8 | Filter: Trials | 334 |
