# Supplementary material for: Ketogenic diet therapies for the treatment of drug-resistant epilepsy in children and adults: A systematic review
Source: PLoS One. 2026 Jul 2;21(7):e0333334. doi: 10.1371/journal.pone.0333334 (PMC13327284; doi:10.1371/journal.pone.0333334)
Supplement: Attachment — Submitted filename: Reviewer response letter.docx [file pone.0333334.s003.docx]

Dear reviewers:

We appreciate your comments and very valuable inputs to our work. We hereby submit our responses below:

**General Comments:**

1. *Please ensure that your manuscript meets PLOS ONE's style requirements, including those for file naming.*

Thank you for the information, the manuscript has been checked and adapted to the requirements.

2. *Thank you for stating the following in the Funding Section of your manuscript: “This work was supported by Advanced Medical Nutrition (AMN), whose representatives had no influence on any stage of the research, nor on the presentation or publication of the results. The authors take full responsibility for the published findings.” We note that you have provided funding information that is currently declared in your Funding Statement. However, funding information should not appear in the Acknowledgments section or other areas of your manuscript. We will only publish funding information present in the Funding Statement section of the online submission form.*

*Please remove any funding-related text from the manuscript and let us know how you would like to update your Funding Statement. Currently, your Funding Statement reads as follows:*

*“This work was supported by Advanced Medical Nutrition (AMN), whose representatives had no influence on any stage of the research, nor on the presentation or publication of the results. The authors take full responsibility for the published findings.”*

*Please include your amended statements within your cover letter; we will change the online submission form on your behalf.*

We have removed all funding information from the manuscript and added the correct statement into the cover letter.

3. *Please include captions for your Supporting Information files at the end of your manuscript, and update any in-text citations to match accordingly. Please see our Supporting Information guidelines for more information:* [*http://journals.plos.org/plosone/s/supporting-information*](http://journals.plos.org/plosone/s/supporting-information)*.*

Thank you, Supporting Information has been adaptaed accordingly.

**Reviewer # 1**

1. *This systematic review evaluates the effectiveness and safety of ketogenic diets (KDs) for drug-resistant epilepsy in children, and adults/adolescents. The review is well-structured and uses appropriate methodologies. The review followed the PRISMA statement and registered a protocol in PROSPERO. The search strategy was comprehensive, including multiple databases and was updated. The methodological quality of the included studies was assessed using the Cochrane Risk of Bias 2 tool, and the certainty of the evidence was evaluated using the GRADE approach.*

We appreciate your comments.

2. *The overall certainty of evidence is moderate for seizure reduction but low to very low for other outcomes, primarily due to a high risk of bias (lack of blinding and potential for co-interventions), imprecision, and inconsistency among studies.*

Thank you for your comment. This information is included in line 311.

3. *The impact of KDs on adverse effects in adults and adolescents remains uncertain. The data on adverse effects in children show no significant differences compared to usual care, although the certainty is low. Adherence to the diet may be slightly lower, but results are inconsistent. Studies on quality of life, cognitive, and behavioral outcomes are scarce, heterogeneous, and of very low certainty. No evidence was found regarding hospitalization rates, status epilepticus, or overall survival.*

Thank you for your comment. This information is included in line 294.

4. *While impossible to blind the patients to a change in diet, the Discussion section could be strengthened by offering a brief perspective on how future randomized controlled trials (RCTs) could more effectively mitigate detection bias.*

Thank you for your comment, we have added this information in line 308.

**Reviewer #2:**

1. *The inclusion criteria specify a minimum follow-up of 28 days for randomized controlled trials. This duration appears relatively short for assessing meaningful changes in seizure frequency, treatment adherence, and adverse effects in epilepsy. Please provide a clear rationale for selecting 28 days as the minimum follow-up period, particularly given that the included studies reportedly had follow-up durations ranging from 6 to 24 months.*

Thank you for your comment. This decision was prespecified in the protocol registered in PROSPERO (CRD42024516319). The rationale for selecting a minimum follow-up of 28 days was primarily based on our a priori expectation that the available evidence might be limited; therefore, we aimed to maintain a sensitive search strategy, particularly to capture studies reporting qualitative outcomes such as quality of life. In addition, we adopted the same eligibility criteria used in the previous Cochrane review (McGill 2020) in order to facilitate an update of the existing evidence base. However, none of the included studies had a follow-up as short as 28 days; the shortest follow-up among the included trials was 2 months.

2. *The objective states that the review included “clinical studies,” whereas the methods indicate that only randomized controlled trials were included. For clarity and accuracy, the objective should explicitly state that only randomized controlled trials were considered.*

Thank you for your comment, this has been changed in line 34

3. *The manuscript reports the use of ICEMAN to assess the credibility of subgroup effects. However, ICEMAN evaluates effect modification rather than overall clinical or statistical heterogeneity across studies. Please clarify how heterogeneity was assessed more broadly, including whether statistical heterogeneity was evaluated and how clinical and methodological variability (diet types, age groups, epilepsy syndromes) was addressed in the synthesis.*

Thank you for your comment. We agree that ICEMAN is specifically designed to assess the credibility of subgroup effects (effect modification) rather than overall heterogeneity across studies. As stated in line 191, we used the ICEMAN tool to assess the possible modifying effect of age. In our review, statistical heterogeneity was evaluated using the I² statistic and the chi-square (Q) test within the meta-analyses. We added this information in line 159.

4. *Add the selection of studies in methodology (PRISMA Flow diagram).*

Thank you for your comment. As defined in the PRISMA statement, criteria for selection of studies are included in line 116 and results from the PRISMA flow diagram are reported in line 166.

5. *The age categorization requires clarification. Children are defined as up to 12 years of age, while adolescents are described as starting from 12 years onward, creating a potential overlap at age 12. Please clarify how participants aged 12 years were categorized. In addition, please specify the overall minimum and maximum ages of participants included across the trials to provide a clearer understanding of the study population.*

Thank you for your comment. Age criteria has been updated in line 42. Information on age range has been added to Table 1.

6. Table 1 provides study-level characteristics of the included trials, While this is useful, the review would also benefit from a summary of participant-level characteristics that are clinically relevant in epilepsy, such as min and max ages, sex distribution, baseline seizure frequency, type and duration of epilepsy, any prior treatments, and, if available, socioeconomic status or ethnicity included in the RCT. Including these details would improve clarity regarding the populations studied and help interpret the applicability of the findings.

Thank you for this helpful suggestion. The information regarding age has now been added. While we agree that participant-level characteristics such as age range, sex distribution, baseline seizure frequency, epilepsy type and duration, prior treatments, and other demographic factors are clinically important, it would not be feasible to include all of these details in Table 1. Table 1 was designed to summarize the key study-level characteristics required for the meta-analysis. Additional participant-level information should therefore be consulted in the full text of the respective studies.

**Additional changes made for language clarity:**

1. *The term “adverse effects” was replaced by “side effects”*
2. *The term Ketogenic Diets was replaced by “Ketogenic Diet Therapies”*
3. *In order to make the article easy to read we avoided the repetition for “lack of blinding” in each outcome. Instead, we mention this in the risk of bias assessment and in the SoF table for each outcome.*
