# Supplementary material for: Ketogenic diet therapies for the treatment of drug-resistant epilepsy in children and adults: A systematic review
Source: PLoS One. 2026 Jul 2;21(7):e0333334. doi: 10.1371/journal.pone.0333334 (PMC13327284; doi:10.1371/journal.pone.0333334)
Supplement: Peer review history

# Peer review: Decision Letter 0

**DOI:** 10.1371/journal.pone.0333334.r001

**Authors:** Sidra Kaleem Jafri

25 Feb 2026

PONE-D-25-49633

Ketogenic Diets for the Treatment of Drug-Resistant Epilepsy in Children and Adults: A systematic review

PLOS One

Dear Dr. Balaciano,

Thank you for submitting your manuscript to PLOS ONE. After careful consideration, we feel that it has merit but does not fully meet PLOS ONE’s publication criteria as it currently stands. Therefore, we invite you to submit a revised version of the manuscript that addresses the points raised during the review process.

Please submit your revised manuscript by Apr 11 2026 11:59PM. If you will need more time than this to complete your revisions, please reply to this message or contact the journal office at plosone@plos.org. When you're ready to submit your revision, log on to https://www.editorialmanager.com/pone/ and select the 'Submissions Needing Revision' folder to locate your manuscript file.

Please include the following items when submitting your revised manuscript:

- A letter that responds to each point raised by the academic editor and reviewer(s). You should upload this letter as a separate file labeled 'Response to Reviewers'.
- A marked-up copy of your manuscript that highlights changes made to the original version. You should upload this as a separate file labeled 'Revised Manuscript with Track Changes'.
- An unmarked version of your revised paper without tracked changes. You should upload this as a separate file labeled 'Manuscript'.

-->If you would like to make changes to your financial disclosure, please include your updated statement in your cover letter. Guidelines for resubmitting your figure files are available below the reviewer comments at the end of this letter.

If applicable, we recommend that you deposit your laboratory protocols in protocols.io to enhance the reproducibility of your results. Protocols.io assigns your protocol its own identifier (DOI) so that it can be cited independently in the future. For instructions see: https://journals.plos.org/plosone/s/submission-guidelines#loc-laboratory-protocols. Additionally, PLOS ONE offers an option for publishing peer-reviewed Lab Protocol articles, which describe protocols hosted on protocols.io. Read more information on sharing protocols at https://plos.org/protocols?utm_medium=editorial-email&utm_source=authorletters&utm_campaign=protocols.

We look forward to receiving your revised manuscript.

Kind regards,

Sidra Kaleem Jafri

Academic Editor

PLOS One

Journal requirements:

When submitting your revision, we need you to address these additional requirements.

1. Please ensure that your manuscript meets PLOS ONE's style requirements, including those for file naming. The PLOS ONE style templates can be found at

https://journals.plos.org/plosone/s/file?id=wjVg/PLOSOne_formatting_sample_main_body.pdf and

https://journals.plos.org/plosone/s/file?id=ba62/PLOSOne_formatting_sample_title_authors_affiliations.pdf

2. Thank you for stating the following in the Funding Section of your manuscript:

“This work was supported by Advanced Medical Nutrition (AMN), whose representatives had no influence on any stage of the research, nor on the presentation or publication of the results. The authors take full responsibility for the published findings.”

We note that you have provided funding information that is currently declared in your Funding Statement. However, funding information should not appear in the Acknowledgments section or other areas of your manuscript. We will only publish funding information present in the Funding Statement section of the online submission form.

Please remove any funding-related text from the manuscript and let us know how you would like to update your Funding Statement. Currently, your Funding Statement reads as follows:

“This work was supported by Advanced Medical Nutrition (AMN), whose representatives had no influence on any stage of the research, nor on the presentation or publication of the results. The authors take full responsibility for the published findings.”

Please include your amended statements within your cover letter; we will change the online submission form on your behalf.

3. Please include captions for your Supporting Information files at the end of your manuscript, and update any in-text citations to match accordingly. Please see our Supporting Information guidelines for more information: http://journals.plos.org/plosone/s/supporting-information.

If the reviewer comments include a recommendation to cite specific previously published works, please review and evaluate these publications to determine whether they are relevant and should be cited. There is no requirement to cite these works unless the editor has indicated otherwise.

Please review your reference list to ensure that it is complete and correct. If you have cited papers that have been retracted, please include the rationale for doing so in the manuscript text, or remove these references and replace them with relevant current references. Any changes to the reference list should be mentioned in the rebuttal letter that accompanies your revised manuscript. If you need to cite a retracted article, indicate the article’s retracted status in the References list and also include a citation and full reference for the retraction notice.

[Note: HTML markup is below. Please do not edit.]

Reviewers' comments:

Reviewer's Responses to Questions

**Comments to the Author**

1. Is the manuscript technically sound, and do the data support the conclusions?

The manuscript must describe a technically sound piece of scientific research with data that supports the conclusions. Experiments must have been conducted rigorously, with appropriate controls, replication, and sample sizes. The conclusions must be drawn appropriately based on the data presented.

Reviewer #1: Yes

Reviewer #2: Yes

**********

2. Has the statistical analysis been performed appropriately and rigorously?

Reviewer #1: Yes

Reviewer #2: Yes

**********

3. Have the authors made all data underlying the findings in their manuscript fully available?

The PLOS Data policy requires authors to make all data underlying the findings described in their manuscript fully available without restriction, with rare exception (please refer to the Data Availability Statement in the manuscript PDF file). The data should be provided as part of the manuscript or its supporting information, or deposited to a public repository. For example, in addition to summary statistics, the data points behind means, medians and variance measures should be available. If there are restrictions on publicly sharing data—e.g. participant privacy or use of data from a third party—those must be specified.

Reviewer #1: Yes

Reviewer #2: Yes

**********

4. Is the manuscript presented in an intelligible fashion and written in standard English?

PLOS ONE does not copyedit accepted manuscripts, so the language in submitted articles must be clear, correct, and unambiguous. Any typographical or grammatical errors should be corrected at revision, so please note any specific errors here.

Reviewer #1: Yes

Reviewer #2: Yes

**********

5. Review Comments to the Author

Please use the space provided to explain your answers to the questions above. You may also include additional comments for the author, including concerns about dual publication, research ethics, or publication ethics. (Please upload your review as an attachment if it exceeds 20,000 characters)

Reviewer #1: This systematic review evaluates the effectiveness and safety of ketogenic diets (KDs) for drug-resistant epilepsy in children, and adults/adolescents. The review is well-structured and uses appropriate methodologies. The review followed the PRISMA statement and registered a protocol in PROSPERO. The search strategy was comprehensive, including multiple databases and was updated. The methodological quality of the included studies was assessed using the Cochrane Risk of Bias 2 tool, and the certainty of the evidence was evaluated using the GRADE approach.

Comments

• The overall certainty of evidence is moderate for seizure reduction but low to very low for other outcomes, primarily due to a high risk of bias (lack of blinding and potential for co-interventions), imprecision, and inconsistency among studies.

• The impact of KDs on adverse effects in adults and adolescents remains uncertain. The data on adverse effects in children show no significant differences compared to usual care, although the certainty is low. Adherence to the diet may be slightly lower, but results are inconsistent. Studies on quality of life, cognitive, and behavioral outcomes are scarce, heterogeneous, and of very low certainty. No evidence was found regarding hospitalization rates, status epilepticus, or overall survival.

• While impossible to blind the patients to a change in diet, the Discussion section could be strengthened by offering a brief perspective on how future randomized controlled trials (RCTs) could more effectively mitigate detection bias.

Reviewer #2: 1) The inclusion criteria specify a minimum follow-up of 28 days for randomized controlled trials. This duration appears relatively short for assessing meaningful changes in seizure frequency, treatment adherence, and adverse effects in epilepsy. Please provide a clear rationale for selecting 28 days as the minimum follow-up period, particularly given that the included studies reportedly had follow-up durations ranging from 6 to 24 months.

2) The objective states that the review included “clinical studies,” whereas the methods indicate that only randomized controlled trials were included. For clarity and accuracy, the objective should explicitly state that only randomized controlled trials were considered.

3) The manuscript reports the use of ICEMAN to assess the credibility of subgroup effects. However, ICEMAN evaluates effect modification rather than overall clinical or statistical heterogeneity across studies. Please clarify how heterogeneity was assessed more broadly, including whether statistical heterogeneity was evaluated and how clinical and methodological variability (diet types, age groups, epilepsy syndromes) was addressed in the synthesis.

4) Add the selection of studies in methodology (PRISMA Flow diagram).

5) The age categorization requires clarification. Children are defined as up to 12 years of age, while adolescents are described as starting from 12 years onward, creating a potential overlap at age 12. Please clarify how participants aged 12 years were categorized. In addition, please specify the overall minimum and maximum ages of participants included across the trials to provide a clearer understanding of the study population.

6) Table 1 provides study-level characteristics of the included trials, While this is useful, the review would also benefit from a summary of participant-level characteristics that are clinically relevant in epilepsy, such as min and max ages, sex distribution, baseline seizure frequency, type and duration of epilepsy, any prior treatments, and, if available, socioeconomic status or ethnicity included in the RCT. Including these details would improve clarity regarding the populations studied and help interpret the applicability of the findings.

**********

6. PLOS authors have the option to publish the peer review history of their article (what does this mean?). If published, this will include your full peer review and any attached files.

If you choose “no”, your identity will remain anonymous but your review may still be made public.

**Do you want your identity to be public for this peer review?**  For information about this choice, including consent withdrawal, please see our Privacy Policy.

Reviewer #1: No

Reviewer #2: **Yes:** HIRA FAROOQ

**********

[NOTE: If reviewer comments were submitted as an attachment file, they will be attached to this email and accessible via the submission site. Please log into your account, locate the manuscript record, and check for the action link "View Attachments". If this link does not appear, there are no attachment files.]

To ensure your figures meet our technical requirements, please review our figure guidelines: https://journals.plos.org/plosone/s/figures

You may also use PLOS’s free figure tool, NAAS, to help you prepare publication quality figures: https://journals.plos.org/plosone/s/figures#loc-tools-for-figure-preparation.

NAAS will assess whether your figures meet our technical requirements by comparing each figure against our figure specifications.

# Author comment: Author response to Decision Letter 1

**DOI:** 10.1371/journal.pone.0333334.r002

29 May 2026

Dear reviewers:

We appreciate your comments and very valuable inputs to our work. We hereby submit our responses below:

General Comments:

1. Please ensure that your manuscript meets PLOS ONE's style requirements, including those for file naming.

Thank you for the information, the manuscript has been checked and adapted to the requirements.

2. Thank you for stating the following in the Funding Section of your manuscript: “This work was supported by Advanced Medical Nutrition (AMN), whose representatives had no influence on any stage of the research, nor on the presentation or publication of the results. The authors take full responsibility for the published findings.” We note that you have provided funding information that is currently declared in your Funding Statement. However, funding information should not appear in the Acknowledgments section or other areas of your manuscript. We will only publish funding information present in the Funding Statement section of the online submission form.

Please remove any funding-related text from the manuscript and let us know how you would like to update your Funding Statement. Currently, your Funding Statement reads as follows:

“This work was supported by Advanced Medical Nutrition (AMN), whose representatives had no influence on any stage of the research, nor on the presentation or publication of the results. The authors take full responsibility for the published findings.”

Please include your amended statements within your cover letter; we will change the online submission form on your behalf.

We have removed all funding information from the manuscript and added the correct statement into the cover letter.

3. Please include captions for your Supporting Information files at the end of your manuscript, and update any in-text citations to match accordingly. Please see our Supporting Information guidelines for more information: http://journals.plos.org/plosone/s/supporting-information.

Thank you, Supporting Information has been adaptaed accordingly.

Reviewer # 1

1. This systematic review evaluates the effectiveness and safety of ketogenic diets (KDs) for drug-resistant epilepsy in children, and adults/adolescents. The review is well-structured and uses appropriate methodologies. The review followed the PRISMA statement and registered a protocol in PROSPERO. The search strategy was comprehensive, including multiple databases and was updated. The methodological quality of the included studies was assessed using the Cochrane Risk of Bias 2 tool, and the certainty of the evidence was evaluated using the GRADE approach.

We appreciate your comments.

2. The overall certainty of evidence is moderate for seizure reduction but low to very low for other outcomes, primarily due to a high risk of bias (lack of blinding and potential for co-interventions), imprecision, and inconsistency among studies.

Thank you for your comment. This information is included in line 311.

3. The impact of KDs on adverse effects in adults and adolescents remains uncertain. The data on adverse effects in children show no significant differences compared to usual care, although the certainty is low. Adherence to the diet may be slightly lower, but results are inconsistent. Studies on quality of life, cognitive, and behavioral outcomes are scarce, heterogeneous, and of very low certainty. No evidence was found regarding hospitalization rates, status epilepticus, or overall survival.

Thank you for your comment. This information is included in line 294.

4. While impossible to blind the patients to a change in diet, the Discussion section could be strengthened by offering a brief perspective on how future randomized controlled trials (RCTs) could more effectively mitigate detection bias.

Thank you for your comment, we have added this information in line 308.

Reviewer #2:

1. The inclusion criteria specify a minimum follow-up of 28 days for randomized controlled trials. This duration appears relatively short for assessing meaningful changes in seizure frequency, treatment adherence, and adverse effects in epilepsy. Please provide a clear rationale for selecting 28 days as the minimum follow-up period, particularly given that the included studies reportedly had follow-up durations ranging from 6 to 24 months.

Thank you for your comment. This decision was prespecified in the protocol registered in PROSPERO (CRD42024516319). The rationale for selecting a minimum follow-up of 28 days was primarily based on our a priori expectation that the available evidence might be limited; therefore, we aimed to maintain a sensitive search strategy, particularly to capture studies reporting qualitative outcomes such as quality of life. In addition, we adopted the same eligibility criteria used in the previous Cochrane review (McGill 2020) in order to facilitate an update of the existing evidence base. However, none of the included studies had a follow-up as short as 28 days; the shortest follow-up among the included trials was 2 months.

2. The objective states that the review included “clinical studies,” whereas the methods indicate that only randomized controlled trials were included. For clarity and accuracy, the objective should explicitly state that only randomized controlled trials were considered.

Thank you for your comment, this has been changed in line 34

3. The manuscript reports the use of ICEMAN to assess the credibility of subgroup effects. However, ICEMAN evaluates effect modification rather than overall clinical or statistical heterogeneity across studies. Please clarify how heterogeneity was assessed more broadly, including whether statistical heterogeneity was evaluated and how clinical and methodological variability (diet types, age groups, epilepsy syndromes) was addressed in the synthesis.

Thank you for your comment. We agree that ICEMAN is specifically designed to assess the credibility of subgroup effects (effect modification) rather than overall heterogeneity across studies. As stated in line 191, we used the ICEMAN tool to assess the possible modifying effect of age. In our review, statistical heterogeneity was evaluated using the I² statistic and the chi-square (Q) test within the meta-analyses. We added this information in line 159.

4. Add the selection of studies in methodology (PRISMA Flow diagram).

Thank you for your comment. As defined in the PRISMA statement, criteria for selection of studies are included in line 116 and results from the PRISMA flow diagram are reported in line 166.

5. The age categorization requires clarification. Children are defined as up to 12 years of age, while adolescents are described as starting from 12 years onward, creating a potential overlap at age 12. Please clarify how participants aged 12 years were categorized. In addition, please specify the overall minimum and maximum ages of participants included across the trials to provide a clearer understanding of the study population.

Thank you for your comment. Age criteria has been updated in line 42. Information on age range has been added to Table 1.

6. Table 1 provides study-level characteristics of the included trials, While this is useful, the review would also benefit from a summary of participant-level characteristics that are clinically relevant in epilepsy, such as min and max ages, sex distribution, baseline seizure frequency, type and duration of epilepsy, any prior treatments, and, if available, socioeconomic status or ethnicity included in the RCT. Including these details would improve clarity regarding the populations studied and help interpret the applicability of the findings.

Thank you for this helpful suggestion. The information regarding age has now been added. While we agree that participant-level characteristics such as age range, sex distribution, baseline seizure frequency, epilepsy type and duration, prior treatments, and other demographic factors are clinically important, it would not be feasible to include all of these details in Table 1. Table 1 was designed to summarize the key study-level characteristics required for the meta-analysis. Additional participant-level information should therefore be consulted in the full text of the respective studies.

Additional changes made for language clarity:

1. The term “adverse effects” was replaced by “side effects”

2. The term Ketogenic Diets was replaced by “Ketogenic Diet Therapies”

3. In order to make the article easy to read we avoided the repetition for “lack of blinding” in each outcome. Instead, we mention this in the risk of bias assessment and in the SoF table for each outcome.

Attachment Submitted filename: Reviewer response letter.docx

# Editor report: Decision Letter 1

**DOI:** 10.1371/journal.pone.0333334.r003

**Authors:** Sidra Kaleem Jafri

16 Jun 2026

Ketogenic Diet Therapies for the Treatment of Drug-Resistant Epilepsy in Children and Adults: A systematic review

PONE-D-25-49633R1

Dear Dr. Balaciano,

We’re pleased to inform you that your manuscript has been judged scientifically suitable for publication and will be formally accepted for publication once it meets all outstanding technical requirements.

Within one week, you’ll receive an e-mail detailing the required amendments. When these have been addressed, you’ll receive a formal acceptance letter and your manuscript will be scheduled for publication.

An invoice will be generated when your article is formally accepted. Please note, if your institution has a publishing partnership with PLOS and your article meets the relevant criteria, all or part of your publication costs will be covered. Please make sure your user information is up-to-date by logging into Editorial Manager at Editorial Manager® and clicking the ‘Update My Information' link at the top of the page. For questions related to billing, please contact billing support.

If your institution or institutions have a press office, please notify them about your upcoming paper to help maximize its impact. If they’ll be preparing press materials, please inform our press team as soon as possible -- no later than 48 hours after receiving the formal acceptance. Your manuscript will remain under strict press embargo until 2 pm Eastern Time on the date of publication. For more information, please contact onepress@plos.org.

Kind regards,

Sidra Kaleem Jafri

Academic Editor

PLOS One

Additional Editor Comments (optional):

Reviewers' comments:

# Editor report: Acceptance letter

**DOI:** 10.1371/journal.pone.0333334.r004

**Authors:** Sidra Kaleem Jafri

PONE-D-25-49633R1

PLOS One

Dear Dr. Balaciano,

I'm pleased to inform you that your manuscript has been deemed suitable for publication in PLOS One. Congratulations! Your manuscript is now being handed over to our production team.

At this stage, our production department will prepare your paper for publication. This includes ensuring the following:

* All references, tables, and figures are properly cited

* All relevant supporting information is included in the manuscript submission,

* There are no issues that prevent the paper from being properly typeset

You will receive further instructions from the production team, including instructions on how to review your proof when it is ready. Please keep in mind that we are working through a large volume of accepted articles, so please give us a few days to review your paper and let you know the next and final steps.

Lastly, if your institution or institutions have a press office, please let them know about your upcoming paper now to help maximize its impact. If they'll be preparing press materials, please inform our press team within the next 48 hours. Your manuscript will remain under strict press embargo until 2 pm Eastern Time on the date of publication. For more information, please contact onepress@plos.org.

You will receive an invoice from PLOS for your publication fee after your manuscript has reached the completed accept phase. If you receive an email requesting payment before acceptance or for any other service, this may be a phishing scheme. Learn how to identify phishing emails and protect your accounts at https://explore.plos.org/phishing.

If we can help with anything else, please email us at customercare@plos.org.

Thank you for submitting your work to PLOS ONE and supporting open access.

Kind regards,

PLOS ONE Editorial Office Staff

on behalf of

Dr. Sidra Kaleem Jafri

Academic Editor

PLOS One
